# Inhibition of L‐type voltage‐gated calcium channel‐mediated Ca^2+^ influx suppresses the collective migration and invasion of ameloblastoma

**DOI:** 10.1111/cpr.13305

**Published:** 2022-07-06

**Authors:** Shujin Li, Hyun‐Yi Kim, Dong‐Joon Lee, Sung‐Ho Park, Keishi Otsu, Hidemitsu Harada, Young‐Soo Jung, Han‐Sung Jung

**Affiliations:** ^1^ Division in Anatomy and Developmental Biology, Department of Oral Biology, Taste Research Center, Oral Science Research Center, BK21 FOUR Project Yonsei University College of Dentistry Seoul South Korea; ^2^ NGeneS Inc Ansan‐si Gyeonggi‐do South Korea; ^3^ Department of Oral & Maxillofacial Surgery Yonsei University College of Dentistry Seoul South Korea; ^4^ Division of Developmental Biology and Regenerative Medicine, Department of Anatomy Iwate Medical University Iwate Japan

## Abstract

**Objectives:**

Ameloblastoma (AM) has been known as a benign but locally invasive tumour with high recurrence rates. Invasive behaviour of the AM results in destruction of the adjacent jawbone and the non‐detectable remnants during surgery, interrupting the complete elimination of cancer cells.

**Methods:**

To explore novel targets for the tumour cell invasion, a transcriptomic analysis between AM and odontogenic keratocyst were performed through next‐generation sequencing in detail.

**Results:**

Enrichment of CACNA1C gene (encoding Cav1.2) in AM, a subunit of the L‐type voltage‐gated calcium channel (VGCC) was observed for the first time. The expression and channel activity of Cav1.2 was confirmed by immunostaining and calcium imaging in the patient samples or primary cells. Verapamil, L‐type VGCC blocker revealed suppression of the Ca^2+^‐induced cell aggregation and collective invasion of AM cells in vitro. Furthermore, the effect of verapamil in suppressing AM invasion into the adjacent bone was confirmed through orthotopic xenograft model specifically.

**Conclusion:**

Taken together, Cav1.2 maybe considered to be a therapeutic candidate to decrease the collective migration and invasion of AM.

## Graphical abstract

L‐type voltage‐gated calcium channels (VGCCs) regulate the Ca^2+^ influx in ameloblastoma (AM) cells. The cell aggregation and collective invasion of AM cells were controlled by the VGCC‐mediated Ca^2+^ signal in vitro and in vivo. Verapamil blocks VGCC effectively and suppresses the aggregation and invasion during AM progression.

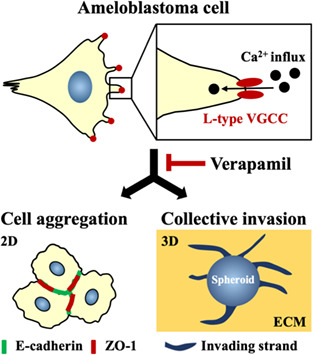

## 1 INTRODUCTION

Ameloblastoma (AM) is one of the most common odontogenic epithelial tumour among all ethnic groups.^1^ Although AM has been classified as a benign tumour, it usually infiltrates into adjacent bone marrow and may penetrate the cortex and invasion into the adjacent muscle and local soft tissues.^2^ The uneliminated cells are considered to be the main cause of the high recurrence rate after conservative surgery, which is up to 90%.^3^ Therefore, the marginal or segmental resection of the jawbone remains the mainstay of management for this disease, although which accompanies severe morbidities including large jawbone defects, impaired oral functions, and facial aesthetic problems. Comprehensive reconstruction of oral tissue is often required after surgical treatment, which remarkably compromises the patient's quality of life and raises healthcare costs.^4^

During the invasion process, multicellular clusters of cancer cells more easily adapt to diverse structural, molecular, and microenvironmental conditions than single cells.^5^ Emerging evidence indicates that collective invasion is frequently observed in solid tumours.^6^, ^7^, ^8^ Various pathological findings demonstrate that conventional AM cells also invade collectively into surrounding tissues without losing the cell–cell adhesion and maintaining epithelial cell characteristics.^9^

Given the indispensable role of the Ca^2+^ signal in various intercellular functions, it is unsurprising that the Ca^2+^ can regulate the cancer progression and numerous signalling transduction. Alternation in the expression or activity of particular Ca^2+^ channels in some cancers promotes cancer progression compared with most normal cells.^10^ Accumulating evidence indicated that the L‐type voltage‐gated calcium channel (VGCC) is involved in many tumorigenic processes ranging from tumour growth, migration, and collective invasion. In brain cancer, the calcium influx through the Cav3.2 (subunit of VGCC) channels trigger the CAMKII‐dependent activation of the p38 MAPK signalling pathway and lead to massive cell growth.^11^ In breast cancer, the application of the L‐type VGCC blocker significantly inhibits the cancer cell migration, and invasion by disrupting the filopodia stabilization and maturation of the focal adhesion.^12^ In head and neck squamous carcinomas, tumour‐derived matrix stiffness and EGFR signalling triggered the intracellular Ca^2+^ influx through Cav1.1 and promote the collective invasion.^13^

To investigate the underlying mechanisms of AM invasion, a transcriptomic comparison between AM and odontogenic keratocyst (OKC) were performed. This is the first report about the *calcium voltage‐gated channel alpha1 C* (*CACNA1C*, coding Cav1.2) is significantly upregulated in the AM compared to OKC. Verapamil, a well‐known L‐type VGCC antagonist,^14^ dramatically suppressed the Cav1.2‐mediated cell aggregation and collective invasion in immortalized or primary AM cells. Taken together, we propose that Cav1.2 could be a potential target for controlling the collective invasion of AM.

## 2 MATERIALS AND METHODS

### 2.1 Patients and surgical specimens

Patients with AM or OKC who successfully underwent surgery at Yonsei University Hospital between 2019 and 2021 were enrolled under the approval of the Institutional Review Boards of Yonsei University Health System (YUHS‐IRB 2‐2018‐0050). Informed consent was provided by all patients enrolled in the study. Specimens from AM (*n* = 8) and OKC (*n* = 8) patients whose diagnoses were confirmed through pathology reports were selected (Table S1). Specimens were separated for the primary cell culture, histological analysis and the RNA sequencing.

### 2.2 RNA sequencing

RNA was extracted from AM (*n* = 3) and OKC (*n* = 3) tissues and mRNA libraries were prepared using the TruSeq Stranded mRNA preparation kit (Illumina). RNA sequencing was performed with Illumina HiSeq2500 sequencing platform for 101‐mer paired‐end reads. FastQC was used for trimming adapter sequences and discarding low quality reads. The reads were mapped on the human reference genome (GRCh37/hg19) and gene expression matrix was generated using TopHat and Cufflinks.^15^ Differentially expressed genes (DEGs) were identified using DEGseq, an R package for DEG analysis, with adjusted *p* < 0.05 and |fold change| > 4 threshold.^16^ Gene ontology (GO) analysis was performed using clusterProfilter, a R package for interpretation of omics data.^17^

### 2.3 Cell culture

The established human AM cell line AM‐1^18^ was cultured in Keratinocyte serum‐free medium (KSFM; 10724‐011; Gibco) supplemented with 2.5 μg EGF Human Recombinant (10450‐013; Gibco), 25 mg bovine pituitary extract (13028‐014; Gibco), and Dulbecco's modified eagle's medium (DMEM; 11995‐065; Gibco) supplemented with 10% foetal bovine serum (C0235; Gibco) and 10,000 U/ml penicillin–streptomycin (15140163; Gibco). AM or OKC tissue enucleated from the patients were dissociated as single cells and cultured following the protocol established previously.^4^ These cells were named as primary AM cells and primary OKC cells (pOKC). All cells were cultured in an incubator (Hera cell; Thermo Fisher Scientific) at 37°C in a humidified atmosphere with 5% carbon dioxide (CO_2_). Cultured cells were used for the following investigations including immunocytochemistry (ICC), Ca^2+^ influx visualization, and cell migration assay.

### 2.4 siRNA transfection

The AM cells were transfected with either negative control siRNA (Scrambled; sc‐398433; Santa Cruz) or CACNA1C siRNA (sc‐42688; Santa Cruz) following the manufacturer's protocol for lipofectamine RNAiMAX (Life Technologies). For the siRNA experiments, 2 × 10^5^ cells were seeded in each six‐well culture dish. Once cells had grown to 80% confluence, a mixture of 4 μl of siRNA (40 pmol/μl) and 6 μl of Lipofectamine 2000 reagent (Invitrogen) was added to the medium of each dish. After 48 h, cells were harvested for the ICC, spheroid invasion and migration assays.

### 2.5 Spheroid formation

AM‐1 or primary AM cells were suspended in DMEM medium and seeded at a density of 2 × 10^4^ cells/well in an ultralow attachment 96‐well plate (Prime Surface 3D Culture Spheroid 96U plates; Sbio). The cells were cultured for 3 days until diameter of spheres became more than 50 μm were counted under the Olympus CKX41SF inverted Polarized Light Microscope (Olympus Corporation) (magnification, ×200). The spheres were used for the invasion assay.

### 2.6 Immunohistochemistry staining

For immunofluorescence staining, the slides were incubated with antibodies against CACNA1C (ACC‐003; Alomone Labs; 1:200), E‐cadherin (610182; BD Biosciences; 1:200), ZO‐1 (ab221547; Abcam; 1:200), vimentin (5714; CST; 1:200), human leukocyte antigen (HLA) (ab70328; Abcam; 1:200), CK14 (ab7800; Abcam; 1:200), and P‐cadherin (ab19350; Abcam; 1:200) at 4°C overnight. Subsequently, secondary antibodies (Invitrogen; 1:200) in PBT for 2 h at room temperature, washed again with PBT, and counterstained with TO‐PRO‐3 (T3605; Thermo Fisher Scientific; 1:1000) in TDW for 15 min. For Phalloidin staining, slides were incubated with Alexa Fluor 488 Phalloidin (A12379; Thermo Fisher Scientific; 1:400). For colorimetric immunohistochemistry of tissues, slides were incubated with antibodies against Ki67 (ab16667; Abcam; 1:200), β‐catenin (sc‐7963; Santa Cruz; 1:100), Axin2 (ab109307; Abcam; 1:200). Subsequently, slides were processed with secondary antibody kit (D03‐110; GBI Labs) and DAB staining kit (C09‐12; GBI Labs) following manufacturer's protocol. All the images were taken by inverted Laser Confocal Microscope (DMi8; Leica).

### 2.7 Time‐lapse imaging

The AM‐1 cell suspensions with a density of 3 × 10^5^ were seeded on a 35‐mm glass bottom confocal dish for at least 24 h. Mix 25 mM Cell Tracker Green CMFDA (Thermo Fisher Scientific) with the medium and incubated in the 37°C incubator for 30 min. These staining methods with fluorescent probe enable us to visualize the AM‐1 cell movement clearly and no effect on cell viability. After washing with PBS three times, confocal dishes with AM‐1 cells in 2 ml of conditional medium were placed into the microscope stage adaptor, and z‐stacks of confocal images were acquired using a spinning disk confocal imaging system based on a CQ‐1 Confocal Quantitative Image Cytometer (CQ‐1; Yokogawa). Then 145 serial two‐dimensional (2D) confocal images through 517 nm channels were recorded in the environmental chamber, which ensured a constant temperature (37°C), humidity and 5% CO_2_ atmosphere throughout the duration of imaging. Intervals between image acquisitions were 10 min. All image acquisition settings were identical for experimental variants in each experiment. ImageJ software package was used for graphical analysis. ImageJ plugins, tracking was used to record the movement trace of AM‐1 cells. Migration distance was calculated by particle analysis of ImageJ. For the calcium imaging, AM‐1 or primary AM cells were transfect with GCamp7s plasmid (Addgene; #104463). The procedure of calcium imaging was followed as previous study.^19^

### 2.8 Spheroid invasion assay

The AM‐1 spheroids were stained with CellTracker RED (Thermo Fisher Scientific) for 20 min in 37°C and harvest from the ultralow attachment 96‐well dish and washing with DPBS (−) twice and cooling in the ice box for 5 min. The hydrogel (rat tail collagen type I; Corning) were prepared according to the product instructions. The collected AM‐1 spheroids were mixed with 4 mg/ml hydrogel, seeding on the confocal dish (SPL) and incubated at 37°C for 30 min. After solidification of the gel the media contain with or without 1.2 mM calcium chloride (Sigma‐Aldrich) were added. Pictures were taken every 15 min for 72 h using CQ‐1 inverted fluorescence microscope.

### 2.9 Establishment of cell line‐based xenograft model

All animal experiments were performed according to the guidelines of the Yonsei University Health System, Intramural Animal Care and Use Committee (YUHS‐IACUC). YUHS‐IACUC complies with the Guide for the care and use of laboratory animals (National Research Council). The animal study plan was reviewed and approved by this committee (2020‐1043). Immunocompromised PN 6 weeks male mice, with bodyweight around 20 g (BALB/C nu/nu purchased from Nara Biotech Co.), were orthotopically implanted with five hydrogel embedded‐spheroids, into the tooth socket after maxillary first molar extraction. Then, to avoid spheroid detach from the tooth socket, the OssGuide (Bioland) were covered. During the tooth extraction and AM‐1 spheroid transplantation procedure, mice were anaesthetised. After 1 week of monitorization, the Verapamil intraperitoneal injection was performed every 48 h for 3 weeks (*n* = 7), and as a mock group (*n* = 7), the PBS was injected. A total of 30 days after inoculation, the mice were sacrificed, and the micro‐CT was performed after sacrifice. The whole maxilla was harvested and fixed in 4% paraformaldehyde (Sangon Biotech Co. Ltd.) at 4°C for 24 h and after dehydration, it was embedded into the paraffin for the histological study.

### 2.10 Statistical analysis

All data are expressed as the mean value ± standard deviation. The Student's *t*‐test and two‐way analysis of variance were performed with measured values using GraphPad Prism 7 software. The *p* < 0.05 was considered statistically significant.

## 3 RESULTS

### 3.1 L‐type VGCC enriched in AM compared to OKC

**FIGURE 1:**
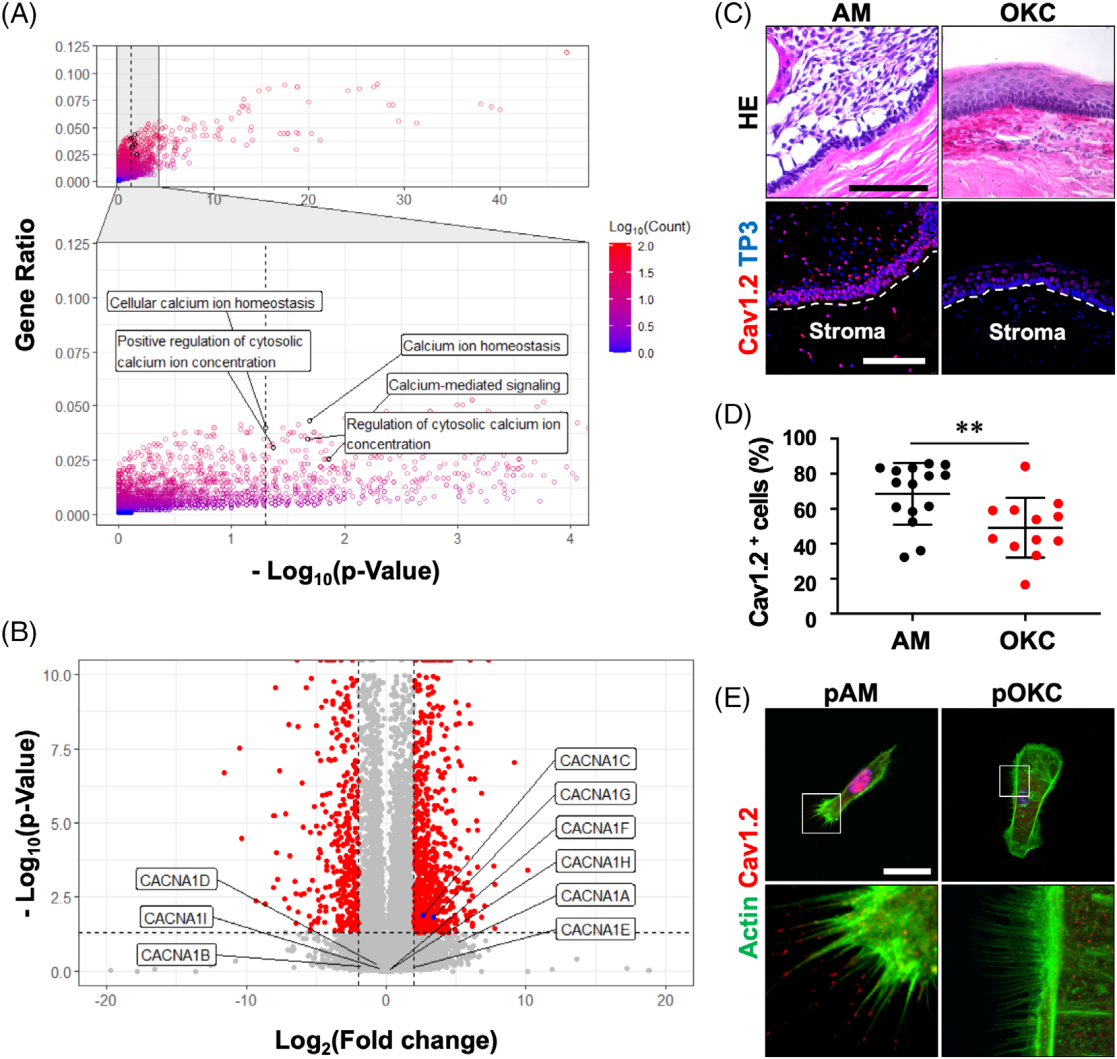
Transcriptomic comparison between ameloblastomas (AMs) and odontogenic keratocysts (OKCs) revealed high expression of Cav1.2 in AMs. (A) Enriched gene ontology terms on upregulated differentially expressed genes (DEGs) in AMs compared with OKCs. Calcium‐related GO terms are specifically labelled. (B) DEGs are visualized using volcano plot, and the DEGs encoding calcium voltage‐gated channel alpha1 subunits are specifically labelled. A horizonal dashed line indicate *p* = 0.05 and vertical dashed lines mark fold change = −4 and 4, respectively. Significant DEGs (*p* < 0.05 and |fold change| > 4) is indicated by red dots. (C) Histopathological structure and Cav1.2 expression in AM and OKC. White dashed lines indicate marginal lines of AM and OKC. (D) Ratio of the Cav1.2‐positive cells per total cells in 15 and 12 sections of AM and OKC tissues are visualized. (E) Immunofluorescence staining of primary AM cells (pAM) and primary OKC cells (pOKC) derived from patients for Actin and Cav1.2. Nuclei were counterstained with TO‐PRO‐3 (TP3). **p < 0.01. Scale bars, C, 100 μm; E, 50 μm.

To identify accessible therapeutic targets against AM, we performed bulk RNA sequencing of three AMs (#1, 2, 3) and three OKC (#1, 2, 3) tissues obtained from individual patients. DEG analysis identified 1042 significantly upregulated and 546 downregulated genes in AMs compared with OKC (Table S2). GO analysis revealed that immune system‐related and keratinization‐related GO terms were enriched in the significantly upregulated and downregulated genes, respectively. Among the 546 significantly enriched GO terms in the upregulated genes, five calcium‐related GO terms were identified (Figure 1A). The five calcium‐related terms shared 10 genes, and among them, *calcium voltage‐gated channel alpha1 C* (*CACNA1C*), a gene encoding Cav1.2, was found (Figure S1). Only *CACNA1C* and *CACNA1G* were significantly upregulated among the nine *calcium voltage‐gated channel alpha1 subunits* (Figure 1B). Cav1.2 was strongly expressed in AM compared to OKC, followed by the percentage of Cav1.2 positive cells significantly increased in AM compared to OKC (Figure 1C,D). In addition, the protein and mRNA expression of Cav1.2 were also validated in other samples (Figures S2 and S3). Previous study has shown that Cav1.2 localized at the tip of filopodia, which promotes cell migration in breast cancer cells.^12^ Similarly, Cav1.2 was expressed at the tips of the filopodia in primary AM cells but not in pOKC, which were obtained from biopsy or resected patients' samples (Figure 1E). And the ratio of Cav1.2 at the tip of filopodia were analysed among three different biological replications (Figure S4).

### 3.2 Specific expression of Cav1.2 and dynamic Ca^2+^ influx in AM‐1 cells

**FIGURE 2:**
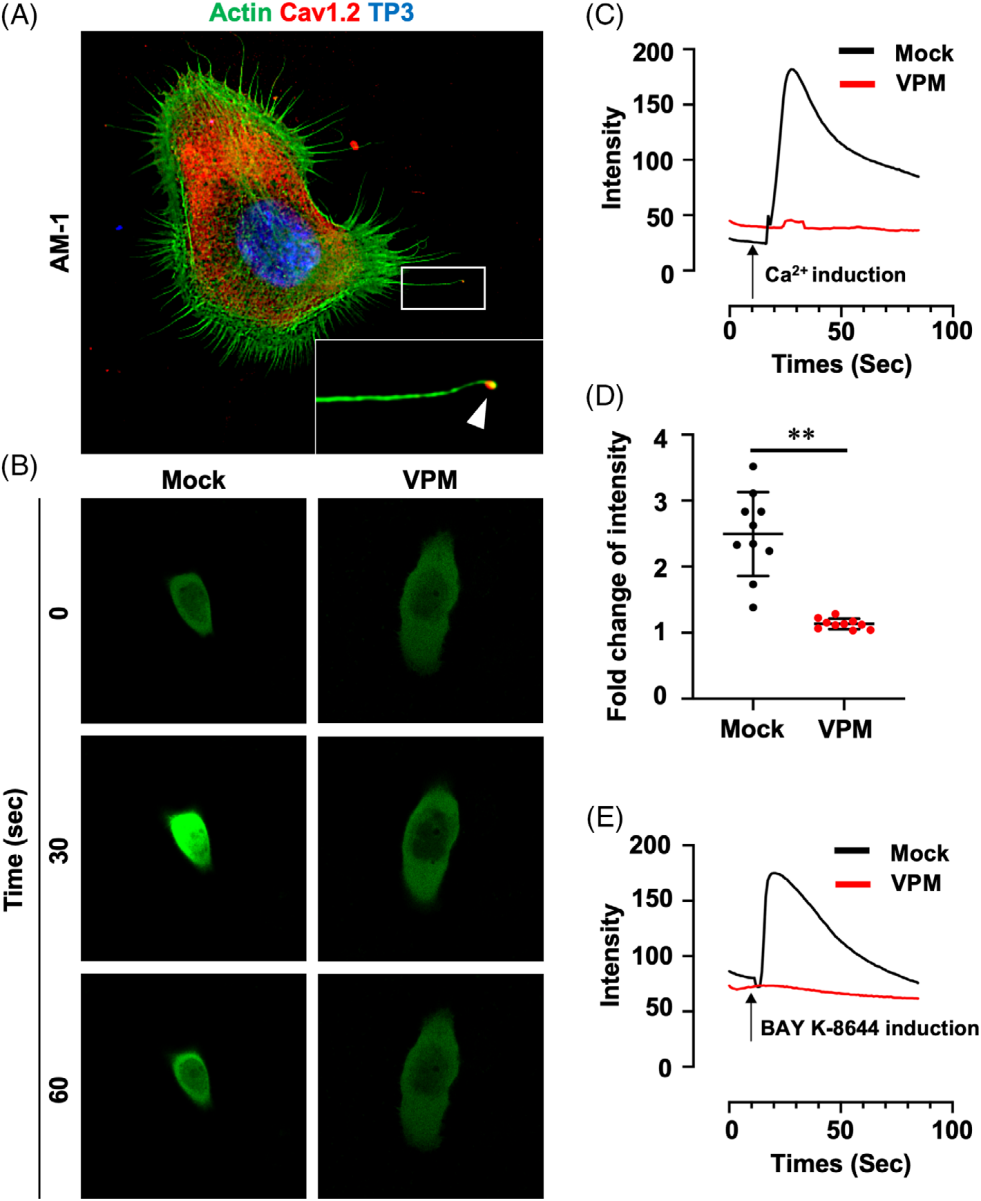
Cav1.2 is expressed and active in immortalized human AM cell line, AM‐1. (A) Immunofluorescence staining of AM‐1 cells for Actin and Cav1.2. Nuclei were counterstained with TO‐PRO‐3 (TP3). (B–E) A green fluorescent protein‐based Ca^2+^ indicator (GCaMP7)‐overexpressed AM‐1 cells were stimulated by calcium chloride or BAY K‐8644 with or without verapamil (VPM). Time‐lapse images (B; Video S1), time‐dependent GFP intensity (C), and fold change of intensity (D, maximum vs. baseline) after calcium chloride treatment with or without VPM. (E) Time‐dependent GFP intensity after BAY K‐8644 treatment with or without VPM. **p < 0.0001. Scale bars, A, 10 μm; B, 20 μm

In order to understand the role of Cav1.2, AM‐1 (AM cell line derived from a human patient) is introduced for further analysis.^18^ Similar to the observation in primary AM cells, Cav1.2 was specifically localized at the tips of filopodia as well as cytoplasm with an intense expression (Figure 2A). To examine the functional activity of Cav1.2, GCaMP7, a green fluorescent protein‐based Ca^2+^ indicator,^20^ was transfected in AM‐1 cells (Video S1). Time‐lapse imaging of the cells revealed transient activation of the Ca^2+^ indicator in response to 0.6 mM Ca^2+^ stimuli (Figure 2B–D, Mock). In the presence of verapamil (an L‐type VGCC antagonist), however, no Ca^2+^ response was observed after induction by the Ca^2+^ stimuli (Figure 2B–D, VPM). Furthermore, BAY K‐8644 (an L‐type VGCC‐specific activator) induction could not stimulated the Ca^2+^ response in the AM‐1 cells with the presence of verapamil (Figure 2E).

### 3.3 Function of L‐type VGCC in cell aggregation and collective migration

**FIGURE 3:**
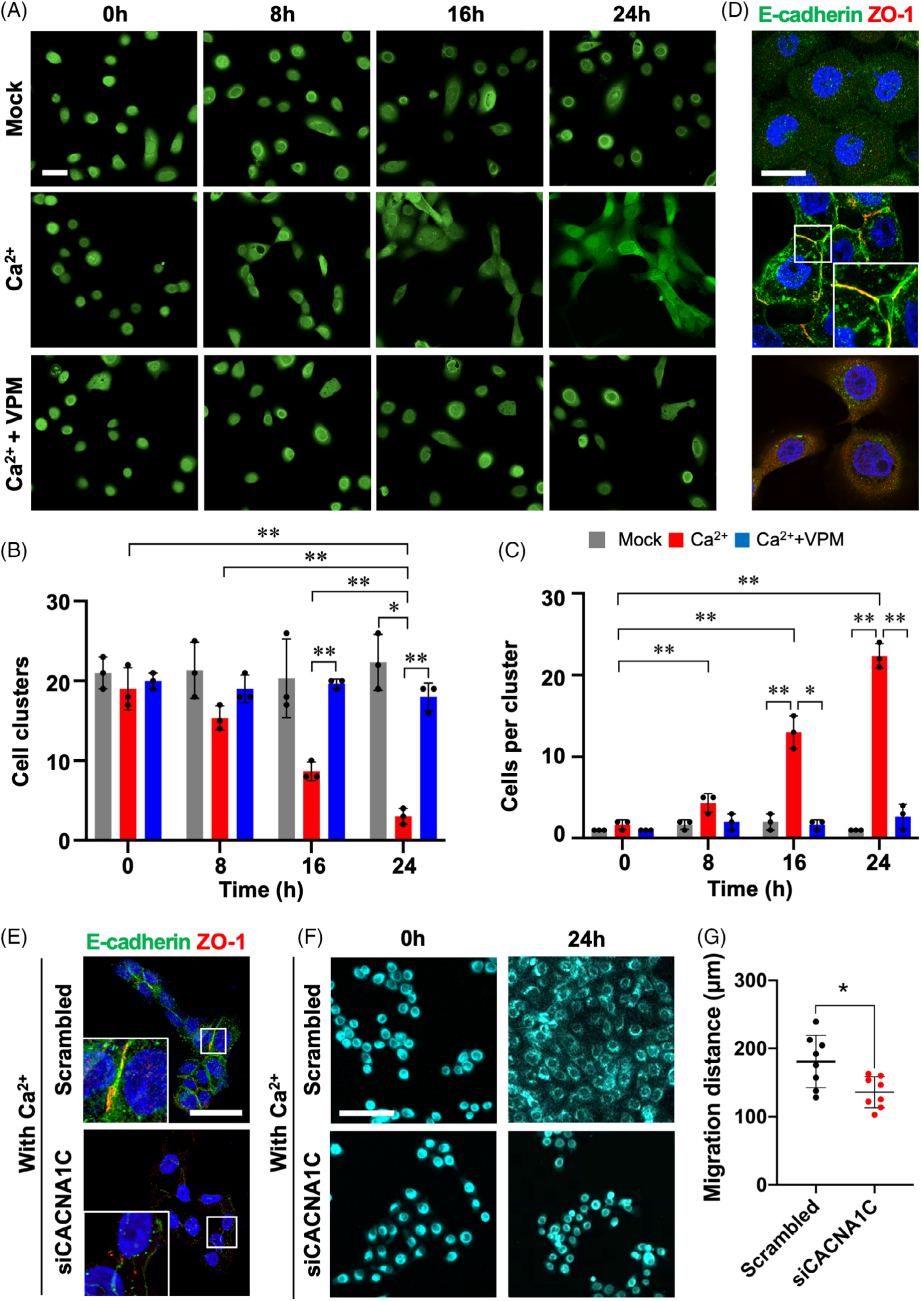
Verapamil suppressed Ca^2+^‐induced cell adhesion and aggregation of AM cells. (A) Time‐lapse images for AM‐1 cells which were stained with a live cell fluorescent dye and treated with or without calcium chloride (Ca^2+^) or verapamil (VPM). Time‐lapse images were combined to video file (Video S2). (B and C) Time‐dependent number of cell clusters (B), and time‐dependent number of cells in each cluster (C) after treatment were visualized and statistically analysed. Student's *t*‐tests were performed for comparing individual values and two‐way ANOVA was performed for comparing groups (two‐way ANOVA reports were presented in Table S3 and S4). (D) Immunofluorescence staining of AM‐1 cells treated without Ca^2+^ or VPM for E‐cadherin and Zonula occludens‐1 (ZO‐1). (E) Immunofluorescence staining of E‐cadherin and ZO‐1 for primary AM cells which were transfected with scrambled or siCACNA1C and culture for 24 h with the presence of Ca^2+^. Nuclei were counterstained with TO‐PRO‐3 (TP3). (F) Time‐lapse images for primary AM cells which were transfected with scrambled or siCACNA1C and stained with a live cell fluorescent dye and culture for 24 h with the presence of Ca^2+^ in the media (Video S3). (G) Quantification of accumulated migration distances of randomly selected individual cells. *p < 0.05, **p < 0.01. Unmarked significance assessment means not significant. Scale bars, A, 50 μm; D, 25 μm; E, 50 μm; F, 100 μm

To investigate the effect of Ca^2+^ and Ca^2+^ channel blockers on collective migration, we performed time‐lapse imaging of AM‐1 cells with or without treatment with Ca^2+^ and verapamil (Figure 3A and Video S2). In media supplemented with Ca^2+^, AM‐1 cells aggregated with each other and formed cell clusters (Figure 3A–C, Ca^2+^). However, verapamil treatment suppressed Ca^2+^‐induced cell aggregation and cluster formation (Figure 3A–C, Ca^2+^ + VPM). ICC results showed that strong expression and junctional accumulation of E‐cadherin and Zonula occludens‐1 (ZO‐1) in Ca^2+^‐supplemented AM‐1 cells (Figure 3D, Ca^2+^). However, the expression and localization of junctional proteins in Ca^2+^ and verapamil co‐treated cells were similar to those in untreated cells (Figure 3D, Mock). To evaluate the role of Cav1.2 in cell aggregation and adhesion, the CACNA1C gene was silenced with siRNA treatment in primary AM cells. The E‐cadherin and ZO‐1 were expressed at the cell to cell junction in the scrambled group, but not expressed in the siCACNA1C group (Figure 3E). Furthermore, the primary AM cells in scrambled group were migrated collectively, whereas individual cell movement was observed in siCACNA1C group (Figure 3F and Video S3). The accumulating migration distance of primary AM cells during 24 h culture (with the presence of Ca^2+^), which were treated with siCACNA1C, were significantly lower than scrambled group (Figure 3G). Taken together, Cav1.2‐mediated intracellular Ca^2+^ is essential for the AM‐1 or primary AM cell aggregation and collective migration.

### 3.4 L‐type VGCC‐mediated Ca^2+^ influx during AM spheroids collective invasion

**FIGURE 4:**
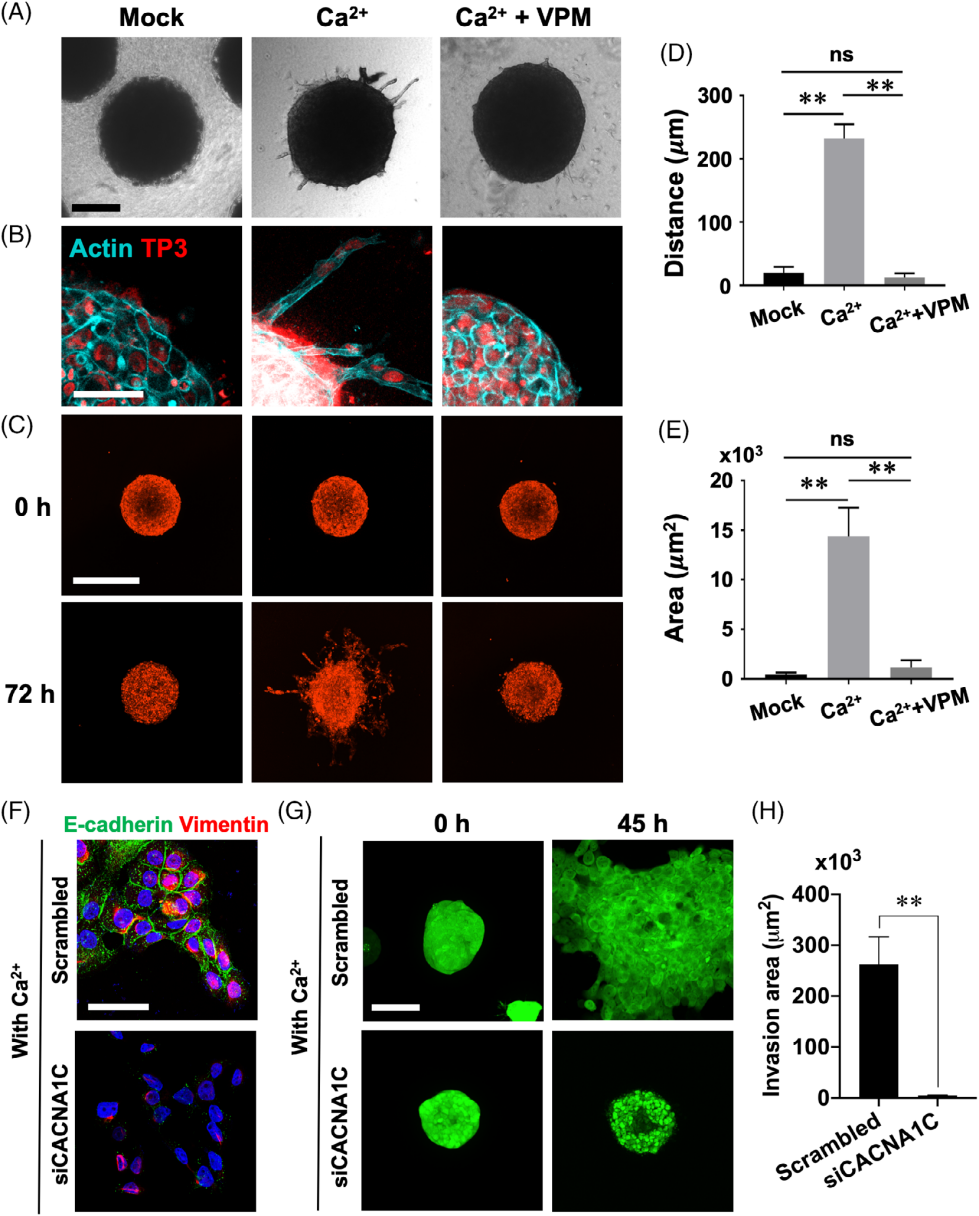
Verapamil inhibited collective invasion of AM spheroid into collagen gel. (A and B) AM‐1 spheroids embed into collagen gel were cultured with or without calcium chloride (Ca^2+^) or verapamil (VPM) for 72 h. Bright field images and cleared whole‐mount staining images for actin. (C) AM‐1 spheroids stained with a live cell fluorescent dye were embed into collagen gel and cultured with or without Ca^2+^ or VPM for 72 h. Time‐lapse images were combined to video file (Video S4). (D and E) Collective invasion distance and area of each spheroid were quantified. The invasion distance and area were measured as differences of maximum distance from spheroid centre or total area between 0 and 72 h. (F) Immunofluorescence staining of E‐cadherin and vimentin for primary AM cells which were transfected with scrambled or siCACNA1C and culture for 24 h with the presence of Ca^2+^. Nuclei were counterstained with TO‐PRO‐3 (TP3). (G) Time‐lapse images for primary AM spheroids which were transfected with scrambled or siCACNA1C and stained with a live cell fluorescent dye (Video S5). (H) Collective invasion area of primary AM spheroids was quantified. The area was measured as differences of maximum distance from spheroid centre or total area between 0 and 45 h. **p < 0.0001; ns, not significant. Scale bars, A, 100 μm; B, 100 μm; C, 500 μm; F, 50 μm; G, 100 μm

In a previous report, we showed that 3D cultured single AM‐1 cells collectively invaded the collagen gel to form a network‐like 3D structure.^21^ To observe the invasion of AM cells more clearly, we performed suspended culture of AM‐1 cells to generate spheroids before embedding into the collagen gel. After 72 h of embedding, collective invasion of the cells into collagen gel without losing cell–cell contacts was clearly observed in the Ca^2+^‐supplemented group (Figure 4A,B, Ca^2+^). However, AM‐1 cells fail to invade into the surrounding collagen gel in the Ca^2+^ and verapamil co‐treated groups (Figure 4A,B, Ca^2+^ + VPM). To visualize the collective invasion of AM‐1 spheroids in a 3D culture condition, time‐lapse images of live cells were taken (Figure 4C and Video S4). The distance from the surface of the spheroid to the end of extrusion (Figure 4D) and invaded area (Figure 4E) demonstrated the suppression of Ca^2+^‐induced invasion by verapamil treatment. Similar results were observed in the Cav1.2 knockdown primary AM cells. The epithelial–mesenchymal transformation (EMT) marker E‐cadherin and vimentin were co‐expressed in the extrusion of the primary AM cell cluster with the presence of Ca^2+^ in the culture media (Figure 4F, scrambled). However, the E‐cadherin was not expressed in the siCACNA1C treated primary AM cells with the presence of Ca^2+^ in the culture media (Figure 4F, siCACNA1C). This result demonstrates that Cav1.2‐mediated Ca^2+^ signal is crucial for the partial EMT in the AM cells. Furthermore, the collective invasion was inhibited in the primary AM cell spheroids with siCACNA1C compared scrambled group (Figure 4G and Video S5). It was also confirmed by the quantification of invasion area (Figure 4H).

**FIGURE 5:**
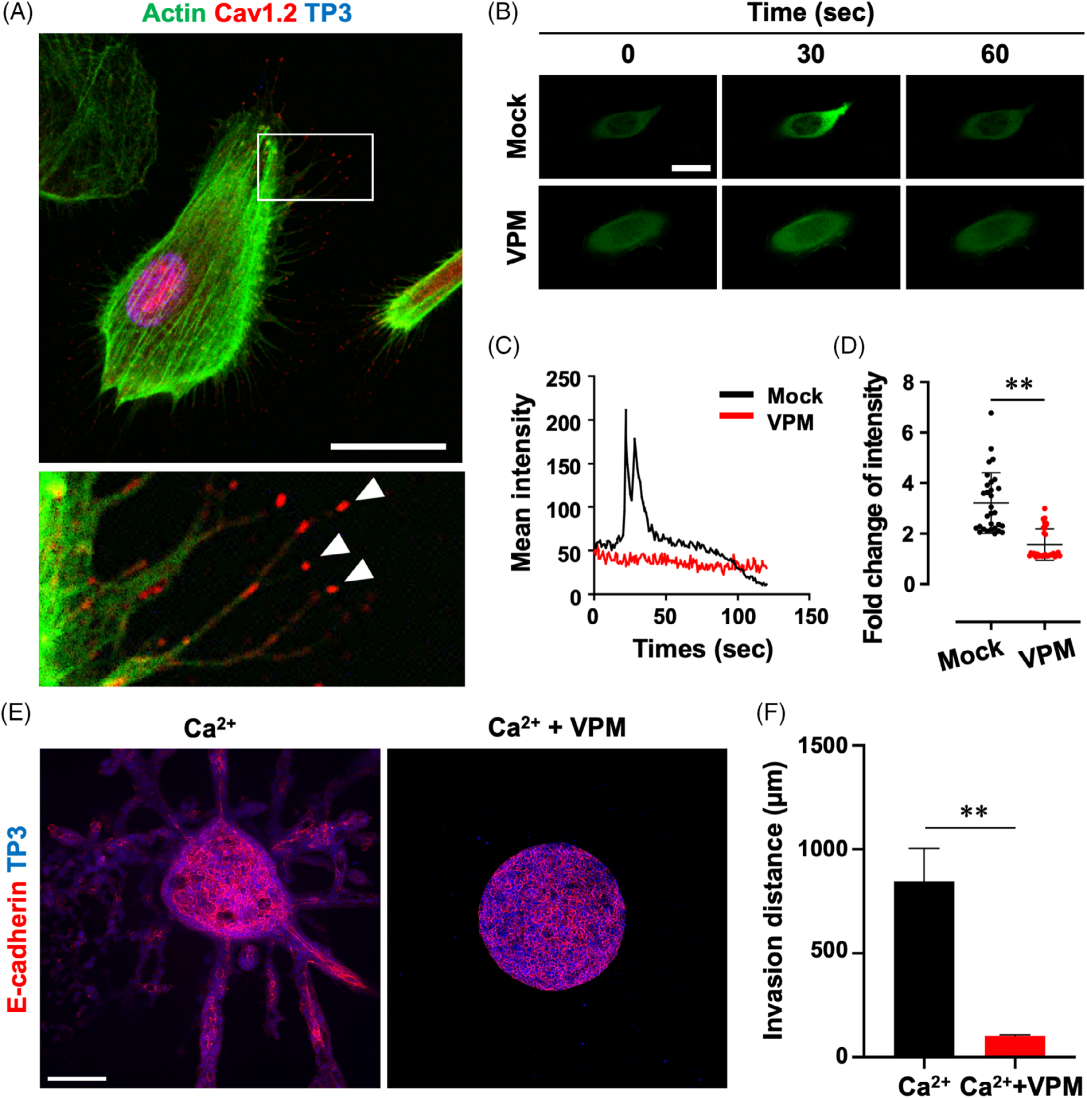
Verapamil suppressed function of Cav1.2 and collective invasion of primary ameloblastoma cells. (A) Immunofluorescence staining of primary AM cells for actin and Cav1.2. (B–D) GCaMP7‐overexpressed primary AM cells were stimulated by calcium chloride (Ca^2+^) with or without verapamil (VPM). Time‐lapse images (B; Video S6), time‐dependent GFP intensity (C), and fold change of intensity (D, maximum vs. baseline) after calcium chloride treatment with or without VPM. (E and F) Primary AM spheroids embed into collagen gel were cultured with or without Ca^2+^ or VPM for 72 h. Cleared whole‐mount staining images for E‐cadherin. Nuclei were counterstained with TO‐PRO‐3 (TP3). Collective invasion of each spheroid was quantified by distance (F) of extrusions. **p < 0.0001. Scale bars, A and B, 20 μm; E, 200 μm

Expression and localization of Cav1.2 were observed at the tips of filopodia of primary AM cells (Figure 5A). Overexpression of GCaMP7 and calcium chloride treatment resulted in a dynamic response of the cell to the Ca^2+^ stimuli and suppression of the response by verapamil treatment (Figure 5B–D and Video S6). In the 3D invasion model, primary AM cells collectively invaded the surrounding collagen gel in Ca^2+^‐supplemented conditions (Figure 5E,F, Ca^2+^). However, the Ca^2+^‐induced invasion of AM cells was suppressed by verapamil treatment (Figure 5E,F, Ca^2+^ + VPM). Overall, L‐type VGCC‐targeted inhibition of Ca^2+^ influx suppressed the collective invasion of AM‐1 or primary AM cell in the in vitro 3D culture system.

### 3.5 Inhibitory effect of verapamil on the AM collective invasion

**FIGURE 6:**
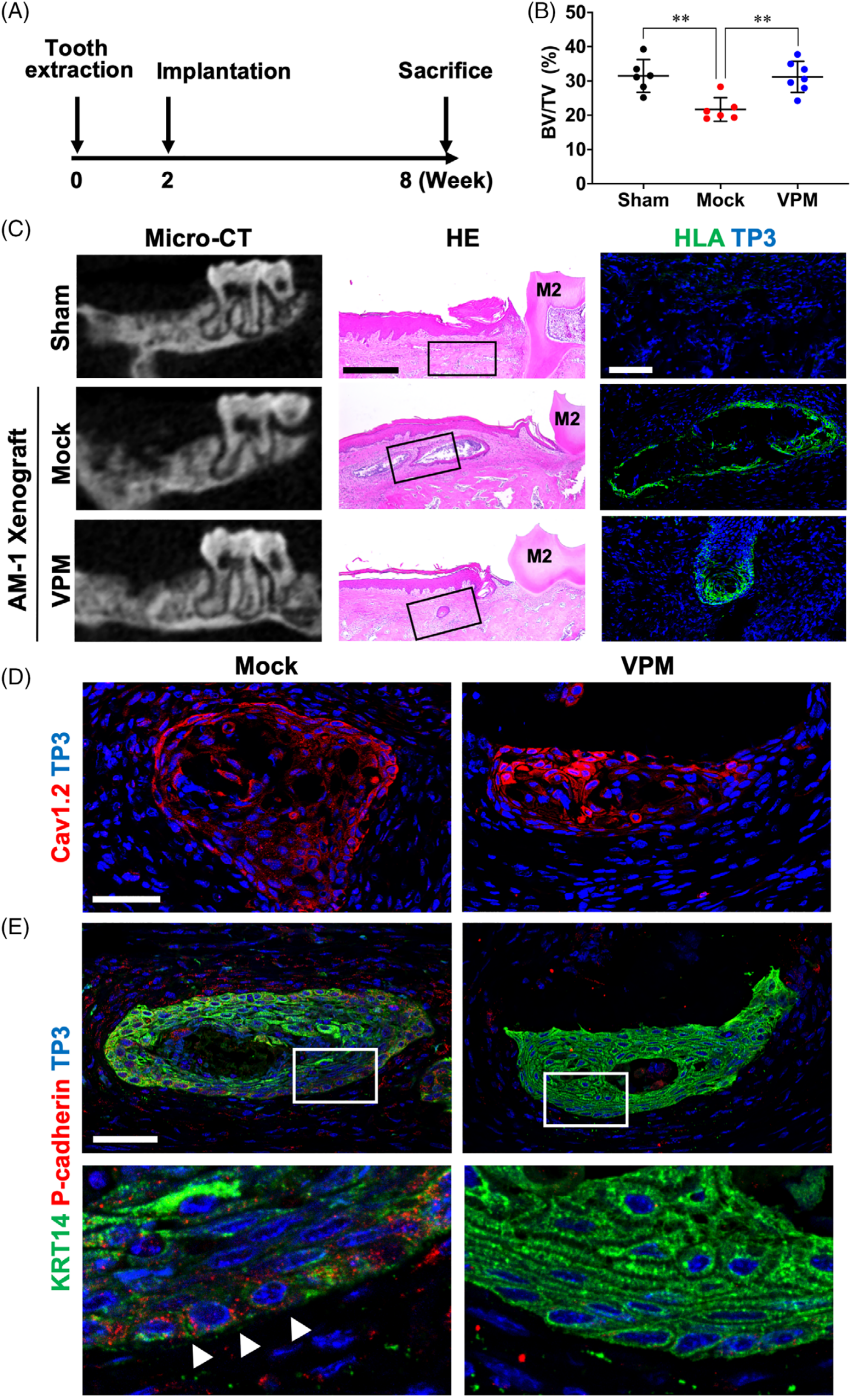
Verapamil suppressed in vivo expansion and invasion of AM‐1 spheroids. (A) Timeline for AM‐1 cell line‐based orthotopic xenograft mouse model. (B) Extraction sites of first right molars in micro‐CT data were set as region of interest and bone volume to total volume (BV/TV) was measured (*n* = 7). (C) Micro‐CT images, haematoxylin and eosin staining (HE), and immunofluorescence staining for human leukocyte antigen (HLA) of maxillae of xenograft mouse models. (D and E) Immunohistochemistry staining for cytokeratin 14 (KRT14), CACNA1C, and P‐cadherin. Nuclei were counterstained with TO‐PRO‐3 (TP3). Scale bars, C, HE, 500 μm; HLA, 100 μm; D, 100 μm; E, 50 μm. **p < 0.0001

To validate the effect of verapamil in vivo, we established an AM‐1 cell line‐based orthotopic xenograft mouse model. The first right molars of the maxilla were extracted and healed for 2 weeks (Figure 6A). Then, the tooth extraction site was drilled and nothing (Sham) or collagen gel‐embedded AM‐1 spheroids (Mock and VPM) were implanted (Figure 6A). In VPM group, 25 mg/kg verapamil was injected every 2 days for 6 weeks (Figure 6A). Then the mice were sacrificed and subjected to radiological, histological, and immunohistological analyses.

Micro‐CT, 3D reconstruction images and Masson's trichrome staining (Figures 6C and S5, Sham) revealed that the tooth socket was filled with alveolar bone in mice without implantation. However, the socket remained empty in the AM‐1 spheroid‐implanted mice (Figures 6C and S5, Mock). The bone volume per total volume (BV/TV) quantification of the socket region also indicated suppression of bone healing by implantation of AM‐1 spheroids (Figure 6B, Mock). Haematoxylin and eosin and HLA expression indicate the cystic structures of epithelial tumour mass in the first molar extraction sites (Figure 6C, Mock and VPM). The expression of Cav1.2 were enriched in the tumour mass in both the Mock and VPM group (Figure 6D). The epithelial structures expressed cytokeratin 14 (KRT14) in an inhomogeneous manner (Figure 6E, Mock) as observed in human AM biopsies.^22^ In specific regions, expression of KRT14 faded into marginal area (Figure 6E, Mock, arrowheads), which implicates presence of EMT events.^22^ Interestingly P‐cadherin, a marker of intermediate/metastable EMT state,^23^ expressed in the marginal area, where the expression of KRT14 faded (Figure 6E, Mock, arrowheads). These results demonstrated that the AM‐1 spheroids survived at the implanted site and invaded into adjacent bone.

In the spheroid‐implanted and verapamil‐injected mice, however, the tooth socket was filled with hard tissue (Figure 6C, VPM) and BV/TV were similar to those in non‐implanted mice (Figure 6B, Sham and VPM). The HLA‐positive cystic structures were also found in the extraction site of the verapamil‐injected mice, but the size was reduced dramatically and most of the space was filled with bone (Figure 6C, VPM). The cystic structures showed strong and homogenous expression of KRT14 and no expression of P‐cadherin (Figure 6E, VPM). Taken together, verapamil suppressed the expansion and invasion of human AM in an orthotropic xenograft mouse model.

## 4 DISCUSSION

Accumulating evidences indicated that several signalling pathway‐related mutations including *BRAF* ^V600E^, *RAS*, *FGFR2*, and *SMO* were synergistically contributed to the AM progression.^24^, ^25^, ^26^, ^27^, ^28^ However, several tentative clinical trials identified that pharmacological inhibition of those relevant signalling pathway exhibited drug resistance or low anticancer efficacy.^29^, ^30^ Dental epithelium, which is considered the normal counterpart of AM, is not available since it degenerates during tooth development.^31^ In the present study, we compared the transcriptome of AM with that of an OKC, a dental epithelium‐originated cyst^32^ which also arising in the jawbone.^33^ A relatively high expression of Cav1.2 in AM compared with OKC were identified, which was validated by immunohistochemical analysis as well. Recently, analyses of available gene expression datasets revealed a wide expression of L‐type VGCCs in diverse cancer cell lines and clinical samples.^34^ As a member of L‐type VGCC, the role of Cav1.1 and Cav1.3 in cancer cell invasion has been investigated in the head and neck cancer or breast cancer, respectively.^12^, ^13^ We propose that Cav1.2 closely associated with the cancer cell invasion and functioning as an oncogenic factor during the AM progression.

Cell motility is involved at every stage of tumorigenesis and contributes to tumour growth, cancer cell migration, and metastasis. Filopodia assembled at the front of invading cancer cells and tightly regulated by the expression of Cav1.3, which has been implicated in breast cancer cell invasion.^12^ The functional activity of L‐type VGCCs were confirmed in immortalized and primary AM cells. Furthermore, Cav1.2 expression was visualized clearly at filopodia tips of AM‐1 and primary AM cells. It suggests an unravelled association between cell motility and Cav1.2 in AM cells.

The cell aggregation and cell adhesion in AM cells were sensitively response to the Ca^2+^ concentration. The administration of verapamil or knockdown of CACNA1C on AM cells exhibited inhibition of cell aggregation and expression of tight junction protein. Cell aggregation and cell–cell adhesion is known as fundamental cellular processes of the collective invasion.^35^ L‐type VGCC‐mediated Ca^2+^ influx (especially through Cav1.2) exhibited regulatory function in AM cell aggregation and cell–cell adhesion which contributes to the process of collective invasion.

To recapitulate the intraosseous tumour microenvironment, AM‐1 cells were 3D cultured in collagen gel or orthotopically implanted into the nude mice's jawbone. We found that suppressing Ca^2+^ influx by verapamil or knockdown of CACNA1C inhibited the collective invasion of AM spheroids in in vitro and in vivo experimental models.

The main conclusion to be drawn is that L‐type VGCC‐mediated Ca^2+^ influx is crucial for the collective migration and invasion of AM. It is also clear that the Cav1.2 could be a potential therapeutic target for suppressing the invasiveness during AM progression. The most urgent need is genetic manipulation studies to provide further insight into the regulatory mechanism of invasiveness through Cav1.2‐mediated Ca^2+^ signalling.

## AUTHOR CONTRIBUTIONS

Shujin Li and Hyun‐Yi Kim contributed to design, data acquisition, analysis, interpretation, and drafted. Dong‐Joon Lee and Sung‐Ho Park contributed to analysis, interpretation, critically revised the manuscript. Keishi Otsu, Hidemitsu Harada, and Young‐Soo Jung contributed to interpretation and critically revised the manuscript. Han‐Sung Jung contributed to conception, and interpretation, critically revised the manuscript. All author gave final approval and agree to be accountable for all aspects of the work.

## CONFLICT OF INTEREST

The authors declare no conflict of interest.

## Supporting information


**Appendix S1** Supporting Information

**Table S2** List of significant differentially expressed genes (DEGs) in ameloblastoma (AM) compared with odontogenic keratocyst (OKC). Separated file named to Table_S2.csv was provided.

**Video S1** A green fluorescent protein‐based Ca^2+^ indicator (GCaMP7)‐overexpressed AM‐1 cells were stimulated by calcium chloride (Ca^2+^) with or without verapamil (VPM).

**Video S2** AM‐1 cells were stained with a live cell fluorescent dye and treated with or without calcium chloride (Ca^2+^) or verapamil (VPM).

**Video S3** Live cell fluorescent dye‐stained primary AM cells treated with scrambled or siCACNA1C and culture for 24 h with the presence of Ca^2+^ in the media.

**Video S4** Live cell fluorescent dye‐stained AM‐1 spheroids were embedded into collagen gel were cultured with or without calcium chloride (Ca^2+^) or verapamil (VPM) for 72 h.

**Video S5** Live cell fluorescent dye‐stained primary AM spheroids were embedded into collagen gel, treated with scrambled or siCACN1C and cultured for 45 h with the presence of Ca^2+^ in the media.

**Video S6** GCaMP7‐overexpressed primary ameloblastoma cells were stimulated by calcium chloride (Ca^2+^) with or without verapamil (VPM).

## Data Availability

The RNA sequencing data have been deposited in the Gene Express Omnibus (GEO) database [GEO: GSE186489].
