# Supplementary material for: Inhibition of L‐type voltage‐gated calcium channel‐mediated Ca2+ influx suppresses the collective migration and invasion of ameloblastoma
Source: Cell Prolif. 2022 Jul 6;55(11):e13305. doi: 10.1111/cpr.13305 (PMC9628225; doi:10.1111/cpr.13305)
Supplement: Supplementary file 1 — Appendix S1 Supporting Information [file CPR-55-e13305-s002.docx]

**Inhibition of L-type voltage-gated calcium channel-mediated Ca^2+^ influx suppresses the collective migration and invasion of ameloblastoma**

**Shujin Li | Hyun-Yi Kim | Dong-Joon Lee | Sung-Ho Park | Keishi Otsu | Hidemitsu Harada | Young-Soo Jung | Han-Sung Jung**

**Supplementary Table, Figures, TABle and Videos**

**Table S1.** List of patient’s information.

|  | Age/sex | Pathological diagnosis | Location |
| --- | --- | --- | --- |
| #1 | 48/M | Ameloblastoma | Mandible |
| #2 | 36/M | Ameloblastoma | Mandible |
| #3 | 56/M | Ameloblastoma | Mandible |
| #4 | 23/F | Ameloblastoma | Rt. Mn. Premolar |
| #5 | 48/M | Ameloblastoma | Rt. Mn. Canine |
| #6 | 26/M | Ameloblastoma | Lt. Mn. Molar |
| #7 | 57/M | Ameloblastoma | Lt. Mn. Molar |
| #8 | 25/M | Ameloblastoma | Lt. Mn. Molar |
| #1 | 42/M | Odontogenic keratocyst | Mandible |
| #2 | 47/M | Odontogenic keratocyst | Mandible |
| #3 | 36/M | Odontogenic keratocyst | Mandible |
| #4 | 40/M | Odontogenic keratocyst | Lt. Mn. Molar |
| #5 | 26/F | Odontogenic keratocyst | Lt. Mn. Molar |
| #6 | 63/F | Odontogenic keratocyst | Rt. Mn. Molar |
| #7 | 67/M | Odontogenic keratocyst | Lt. Mn. Premolar |
| #8 | 54/F | Odontogenic keratocyst | Rt. Mn. Premolar |
|  | | | |

**Table S2.** List of significant differentially expressed genes (DEGs) in ameloblastoma (AM) compared with odontogenic keratocyst (OKC). Separated file named to Table_S2.csv was provided.

**Table S3.** Table of ANOVA results for Figure 3B.

| Table Analyzed | Cell clusters |  |  |  |  |  |
| --- | --- | --- | --- | --- | --- | --- |
|  |  |  |  |  |  |  |
| Two-way RM ANOVA | Matching: Stacked |  |  |  |  |  |
| Assume sphericity? | No |  |  |  |  |  |
| Alpha | 0.05 |  |  |  |  |  |
|  |  |  |  |  |  |  |
| Source of Variation | % of total variation | P value | P value summary | Significant? | Significant? | Significant? |
| Time x Treatment | 24.39 | <0.0001 | **** | Yes | Yes | Yes |
| Time | 13.16 | <0.0001 | **** | Yes | Yes | Yes |
| Treatment | 50.88 | 0.0033 | ** | Yes | Yes | Yes |
| Replicate | 8.939 | <0.0001 | **** | Yes | Yes | Yes |
|  |  |  |  |  |  |  |
| ANOVA table | SS | DF | MS | F (DFn, DFd) | F (DFn, DFd) | F (DFn, DFd) |
| Time x Treatment | 303.3 | 6 | 50.55 | F (6, 18) = 27.71 | F (6, 18) = 27.72 | F (6, 18) = 27.73 |
| Time | 163.6 | 3 | 54.55 | F (1.897, 11.38) = 29.90 | F (1.897, 11.38) = 29.91 | F (1.897, 11.38) = 29.92 |
| Treatment | 632.7 | 2 | 316.4 | F (2, 6) = 17.07 | F (2, 6) = 17.08 | F (2, 6) = 17.09 |
| Replicate | 111.2 | 6 | 18.53 | F (6, 18) = 10.16 | F (6, 18) = 10.17 | F (6, 18) = 10.18 |
| Residual | 32.83 | 18 | 1.824 |  |  |  |
|  |  |  |  |  |  |  |
| Data summary |  |  |  |  |  |  |
| Number of columns (Treatment) | 3 |  |  |  |  |  |
| Number of rows (Time) | 4 |  |  |  |  |  |
| Number of subjects (Replicate) | 9 |  |  |  |  |  |
| Number of missing values | 0 |  |  |  |  |  |

**Table S4.** Table of ANOVA results for Figure 3C.

| Table Analyzed | Cells per cluster |  |  |  |  |
| --- | --- | --- | --- | --- | --- |
|  |  |  |  |  |  |
| Two-way RM ANOVA | Matching: Stacked |  |  |  |  |
| Assume sphericity? | No |  |  |  |  |
| Alpha | 0.05 |  |  |  |  |
|  |  |  |  |  |  |
| Source of Variation | % of total variation | P value | P value summary | Significant? | Geisser-Greenhouse's epsilon |
| Time x Treatment | 35.04 | <0.0001 | **** | Yes |  |
| Time | 20.55 | <0.0001 | **** | Yes | 0.5364 |
| Treatment | 42.59 | <0.0001 | **** | Yes |  |
| Replicate | 0.876 | 0.0431 | * | Yes |  |
|  |  |  |  |  |  |
| ANOVA table | SS | DF | MS | F (DFn, DFd) | P value |
| Time x Treatment | 500.1 | 6 | 83.34 | F (6, 18) = 111.1 | P<0.0001 |
| Time | 293.2 | 3 | 97.73 | F (1.609, 9.656) = 130.3 | P<0.0001 |
| Treatment | 607.7 | 2 | 303.9 | F (2, 6) = 145.9 | P<0.0001 |
| Replicate | 12.5 | 6 | 2.083 | F (6, 18) = 2.778 | P=0.0431 |
| Residual | 13.5 | 18 | 0.75 |  |  |
|  |  |  |  |  |  |
| Data summary |  |  |  |  |  |
| Number of columns (Treatment) | 3 |  |  |  |  |
| Number of rows (Time) | 4 |  |  |  |  |
| Number of subjects (Replicate) | 9 |  |  |  |  |
| Number of missing values | 0 |  |  |  |  |

**
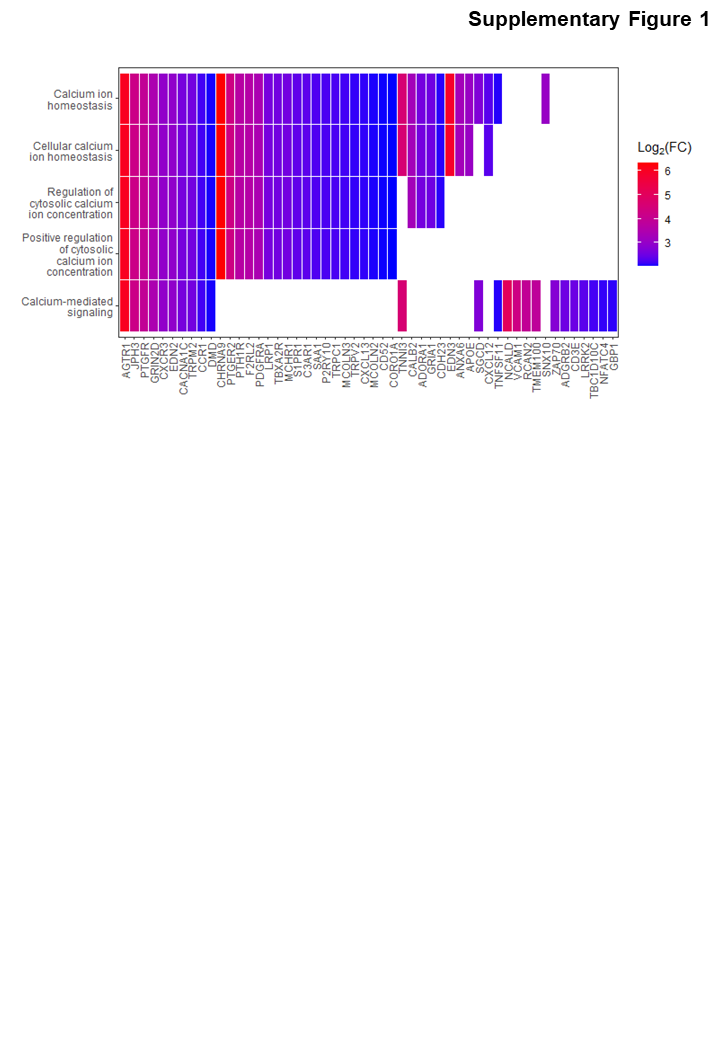
**

**Figure S1.** Calcium-related gene ontology terms (GO) enriched on significantly upregulated DEGs in AM compared with OKC (y axis) and the GO term-related genes were visualized as a heatmap. The color of blocks indicate logarithm to base 2 of fold change (FC).


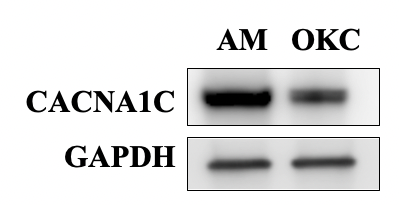


**Figure S2.** Expression of Cav1.2 in AM and OKC samples from patient. The expression of Cav1.2 in AM is significantly higher than OKC.


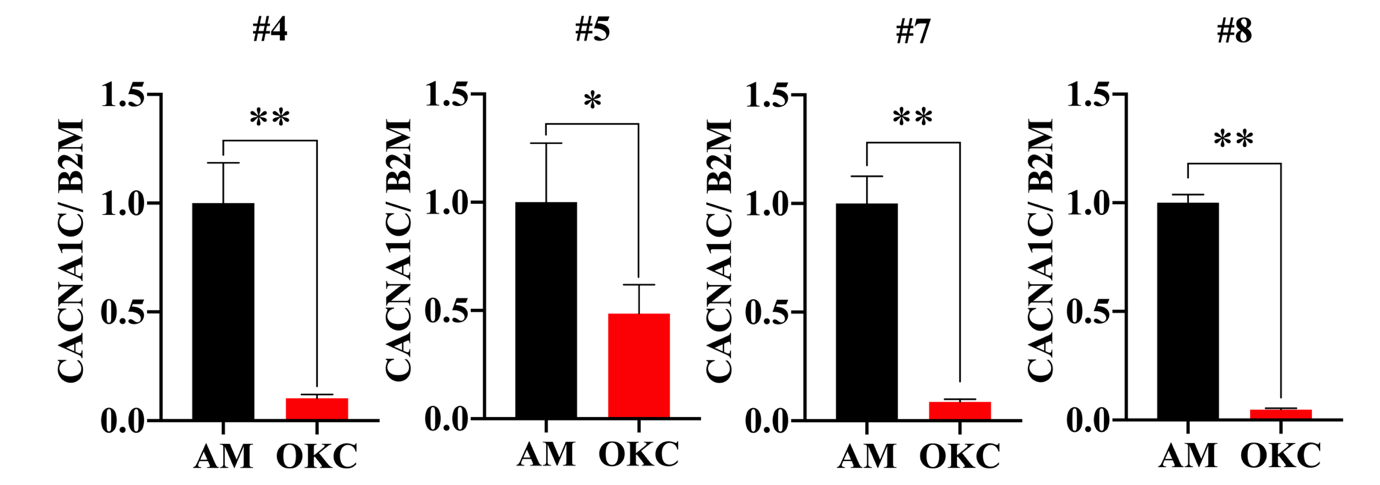


**Figure S3.** Validation of patients’ samples of AM and OKC. Except sample #6, the relative mRNA expression of *CACNA1C* in AM were significantly upregulated in OKC (sample #4, 5, 7, and 8). ** *p*<0.01.


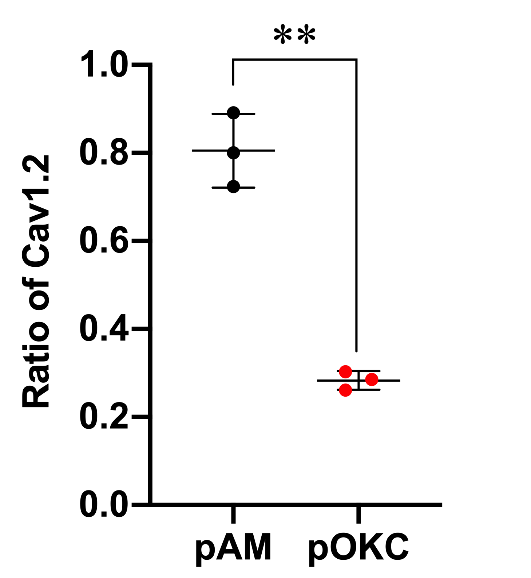


**Figure S4.** Quantification of the ratio of pAM and pOKC cells which express Cav1.2 at the tip of filopodia. The ratio of Cav1.2 at the tip of filopodia were analyzed with confocal image (294 x 294 μm) acquired at three different culturing wells, respectively (biological replication). ** *p*<0.001.


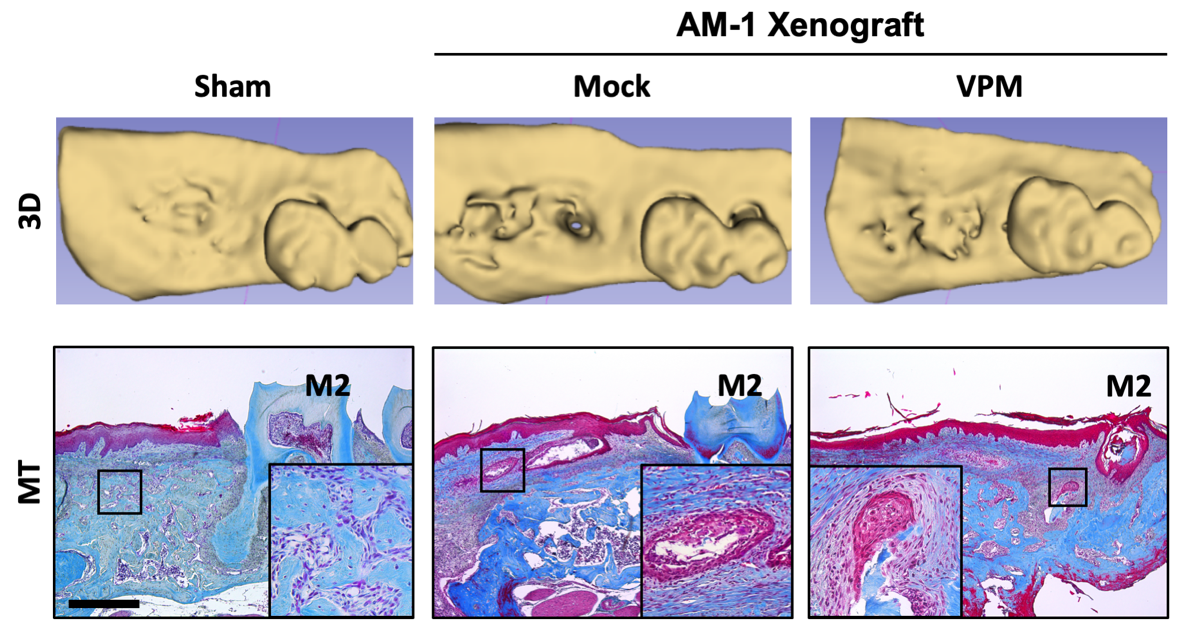


**Figure S5.** The 3D reconstruction images of micro-CT revealed that the tooth socket was filled with alveolar bone in mice without implantation (Sham). The socket remained empty in the AM-1 spheroid-implanted mice (Mock). In the spheroid-implanted and verapamil-injected mice, the tooth socket was filled with hard tissue (VPM). Sections of maxilla were subjected by Masson’s trichrome staining. Scale bar, 500 μm.

**Video S1.** A green fluorescent protein-based Ca^2+^ indicator (GCaMP7)-overexpressed AM-1 cells were stimulated by calcium chloride (Ca^2+^) with or without verapamil (VPM).

**Video S2.** AM-1 cells were stained with a live cell fluorescent dye and treated with or without calcium chloride (Ca^2+^) or verapamil (VPM).

**Video S3.** Live cell fluorescent dye-stained primary AM cells treated with scrambled or siCACNA1C and culture for 24 h with the presence of Ca^2+^ in the media.

**Video S4.** Live cell fluorescent dye-stained AM-1 spheroids were embedded into collagen gel were cultured with or without calcium chloride (Ca^2+^) or verapamil (VPM) for 72 h.

**Video S5.** Live cell fluorescent dye-stained primary AM spheroids were embedded into collagen gel, treated with scrambled or siCACN1C and cultured for 45 h with the presence of Ca^2+^ in the media.

**Video S6.** GCaMP7-overexpressed primary ameloblastoma cells were stimulated by calcium chloride (Ca^2+^) with or without verapamil (VPM).
